# Complete Genome Sequences of Seven EA Cluster Microbacteriophages, Bustleton, MillyPhilly, Riyhil, Phriends, Pherbot, PrincePhergus, and TinSulphur

**DOI:** 10.1128/MRA.01193-19

**Published:** 2019-11-14

**Authors:** Anh M. Nguyen Quach, Amy E. Varghese, Kumal Siddiq, Isabella G. Pappano, Almas Thaha, Tracy A. Marcelis, Bora Feruku, Akhil Vindyala, Riyaz Mohamed, Kailee A. Johnson, Sayema Hakim, Shivashree Sekar, Pavithra Vinnakota, Nowrin H. Borsha, Ryneisha McKenzie, Sanya S. Ailani, Shruti Patel, Vincent A. Beggarly, Drexel University SEA-PHAGES annotators 2019, Matthew T. McDonald, Ritu R. Dalia, Svetlana Khakhina, Susan M. R. Gurney

**Affiliations:** a Department of Biology, Drexel University, Philadelphia, Pennsylvania, USA; Queens College

## Abstract

Seven EA cluster microbacteriophages were isolated from soil collected around Philadelphia, PA, using the bacterial host Microbacterium foliorum. All of these phages have a highly conserved genome with regions of diversity localized to the 3′ end. In phage Phriends (EA1 cluster), this region contains an orpham gene with no known function.

## ANNOUNCEMENT

Bacteriophages are viruses that infect and kill bacteria (1). Phages have been further explored as alternative therapies for antibiotic-resistant bacterial pathogens (2). In this study, we isolated seven cluster EA phages (Table 1) by using Microbacterium foliorum (NRRL B-24224) as a host. *M. foliorum* was originally isolated from grasses in Germany (3), but it is commonly found in human samples and can cause opportunistic infections in hospital settings (4).

**TABLE 1:** Sequencing data for seven cluster EA microbacterium phages

Phage name	Subcluster	GenBank accession no.	SRA accession no.	Genome length (bp)	G+C content (%)	No. of protein-coding genes	No. of tRNA genes (anticodon)	Genome sequencing coverage (×)
Bustleton	EA4	MK814762	SRX6610497	39,184	64.4	58	1 (AAA)	1,137
MillyPhilly	EA1	MK620903	SRX6610496	41,800	63.5	63	0	1,455
Phriends	EA1	MK620902	SRX6610498	41,606	63.6	62	0	1,140
Pherbot	EA4	MK620904	SRX6610499	39,171	64.4	58	1 (AAA)	1,784
PrincePhergus	EA4	MK620901	SRX6610494	39,191	64.6	58	1 (CGG)	1,299
Riyhil	EA1	MK620895	SRX6610493	41,812	63.5	63	0	1,834
TinSulphur	EA1	MK620905	SRX6610495	41,807	63.5	64	0	1,214

Microbacteriophages can currently be grouped into 12 clusters based on genetic similarity (https://phagesdb.org) and further divided into subclusters. Here, we describe four EA1 subcluster phages, namely, MillyPhilly, Riyhil, Phriends, and TinSulphur, and three EA4 subcluster phages, namely Bustleton, Pherbot, and PrincePhergus. All seven phages are lytic Siphoviridae bacteriophages and have various plaque sizes. All these phages were isolated from soil samples collected in Philadelphia and Marshallton (TinSulphur), PA. Phages were isolated by following Enriched Isolation Protocol 5.5 in the Phage Discovery Guide (https://seaphagesphagediscoveryguide.helpdocsonline.com/home), using M. foliorum as a host bacterium and incubating in peptone yeast calcium (PYCa) broth for 96 h at 30°C with shaking. The resulting enriched lysates were tested for the presence of phages using Spot Test Protocol 5.6 in the Phage Discovery Guide. Isolated bacteriophage plaques were purified and amplified, and DNA was extracted using the Promega Wizard DNA cleanup kit. The genomes were individually sequenced with the Illumina MiSeq platform by the Pittsburgh Bacteriophage Institute. Sequencing libraries were prepared from genomic DNA with an NEBNext Ultra II FS kit. A total of 48 libraries were multiplexed and sequenced with an Illumina MiSeq platform, yielding at least 100,000 150-base single-end reads for each genome sequence, sufficient for at least 1,137-fold coverage (Table 1). Raw reads were assembled with Newbler v2.9 with default settings; each assembly yielded a single phage contig, which was checked for completeness, accuracy, and phage genomic termini with Consed v29. After sequencing, the genomes were annotated using the following databases and software: DNA Master v5.23.2 (http://cobamide2.bio.pitt.edu), Glimmer v3.02 (5), GeneMark v2.5p (6), Starterator, Phamerator (https://phamerator.org) (7), PhagesDB BLAST (phagesdb.org/blast), NCBI-BLASTn, NCBI-BLASTp dbv4 (cutoff E value, 10^−14^), the NCBI Conserved Domain Database v3.17 (8, 9), HHPRED v3.2.0 (10) (cutoff E value, 10^−5^), ARAGORN v1.2.38 (11), tRNAscanSE v2.0 (12), and the Phage Evidence Collection and Annotation Network (PECAAN). Default settings were used for each of these programs/databases.

The genome of each of the four EA1 microbacteriophages is 41,606 to 41,812 bp long and has 62 to 64 protein-coding genes (Table 1). Whole-genome nucleotide alignment performed by BLASTn showed highly conserved genomes with between 98.4 and 99% pairwise nucleotide sequence identity shared between the four phages. Phage Phriends contains two rare genes, namely, *gp52*, an orpham (a gene pham which contains only a single member [7]) with no known function, and *gp55*, which was previously found only in EA9 microbacteriophage ChickenKing.

The newly isolated EA4 microbacteriophages have closely related genomes with 96 to 99% pairwise nucleotide sequence identity. The genome lengths vary by less than 20 bp (range, 39,171 to 39,191 bp). Each of these three phages encodes 58 genes, including 1 tRNA gene. Interestingly, the tail assembly chaperones are predicted to be translated using a canonical −1 translational frameshift, whereas in EA1 phages, they are predicted to be translated as separate open reading frames.

### Data availability.

The genome sequences reported here are deposited in GenBank under the accession numbers provided in Table 1. Microbacteriophage ChickenKing has the GenBank accession number MN310543. Sequencing reads were deposited in the Sequence Read Archive (SRA) under SRA accession numbers provided in Table 1 and under BioProject number PRJNA488469.
